# Association of cognition with temporal discounting in community based older persons

**DOI:** 10.1186/1471-2318-12-48

**Published:** 2012-08-31

**Authors:** Patricia A Boyle, Lei Yu, Eisuke Segawa, Robert S Wilson, Aron S Buchman, David I Laibson, David A Bennett

**Affiliations:** 1 Rush University Medical Center, Rush Alzheimer’s Disease Center, 600 S. Paulina, Suite 1020B, Chicago, IL, 60612, USA; 2 Department of Behavioral Sciences, Stanford, USA; 3 Department of Neurological Sciences, Stanford, USA; 4 Department of Economics, Harvard University, Cambridge, USA

**Keywords:** Aging, Cognition, Temporal discounting, Preferences, Decision making

## Abstract

**Background:**

The objective of this study was to test the hypothesis that cognitive function is negatively associated with temporal discounting in old age.

**Methods:**

Participants were 388 community-dwelling older persons without dementia from the Rush Memory and Aging Project, an ongoing longitudinal epidemiologic study of aging in the Chicago metropolitan area. Temporal discounting was measured using standard questions in which participants were asked to choose between an immediate, smaller payment and a delayed, larger one. Cognition was measured using a detailed battery including 19 tests. The association between cognition and temporal discounting was examined via mixed models adjusted for age, sex, education, income, and the number of chronic medical conditions.

**Results:**

Descriptive data revealed a consistent pattern whereby older persons with lower cognitive function were more likely to discount greater but delayed rewards compared to those with higher cognitive function. Further, in a mixed effect model adjusted for age, sex, education, income, and chronic medical conditions, global cognitive function was negatively associated with temporal discounting (estimate = −0.45, SE = 0.18, p = 0.015), such that a person with lower cognition exhibited greater discounting. Finally, in subsequent models examining domain specific associations, perceptual speed and visuospatial abilities were associated with temporal discounting, but episodic memory, semantic memory and working memory were not.

**Conclusion:**

Among older persons without dementia, a lower level of cognitive function is associated with greater temporal discounting. These findings have implications regarding the ability of older persons to make decisions that involve delayed rewards but maximize well-being.

## Background

Most decisions made in everyday life involve choices between immediate and delayed rewards. From seemingly minor choices, such as deciding to pass up mouth-watering French fries in favor of a healthy salad, to larger ones, such as deciding to forego spending money from today’s paycheck to invest it for retirement, opportunities for intertemporal tradeoffs abound. Temporal discounting, the tendency to prefer smaller, immediate rewards over larger, delayed ones, is a concept that has been widely studied in the economics, behavioral economics, neuroscience and psychology literatures and is related to several important real world financial and health behaviors [1-3]. Individuals who discount future rewards tend to spend more, save less, and make poorer investment decisions [4,5]. Further, those who discount tend to under-utilize health insurance yet exercise less, use alcohol and drugs more, engage in unsafe sex, and are more likely to be obese [6-8]. Thus, temporal discounting is associated with a vast array of behaviors that have significant individual and societal-level consequences.

Recognition of the important role of temporal discounting in human behavior has generated interest in identifying the factors associated with it, and data from studies of younger and middle-aged adults have demonstrated that something akin to cognitive ability (e.g., performance on aptitude or achievement tests) is negatively associated with temporal discounting [9-13]. To date, however, little is known about temporal discounting among older persons. Some studies suggest that older persons may discount less steeply than younger persons, although findings are mixed [14-19], but we are not aware of studies that have examined whether cognitive ability is related to temporal discounting among older adults. Considering that aging is a time when some of life’s most significant financial and health decisions are made (*e.g*., asset decumulation, asset allocation, annuitization, intergenerational transfers, Medicare/health insurance choices, end of life decisions), an understanding of the factors associated with discounting may have major financial and public health implications.

In this study, we examined the relation of cognition, related demographic (i.e., age, sex, education) and contextual (i.e., income, chronic medical conditions) factors with temporal discounting among more than 380 older persons without dementia from the Rush Memory and Aging Project [20]. All participants underwent detailed cognitive evaluations and assessments of temporal discounting using standard behavioral economics questions in which participants were asked to choose between an immediate, smaller payment of $10 versus a delayed, larger payment that ranged from $10.75 to $30. The associations of global cognitive function and five specific cognitive abilities with temporal discounting were examined via mixed effect models adjusted for age, sex, education, income, and chronic medical conditions.

## Methods

### Participants

Participants were from the Rush Memory and Aging Project, an ongoing longitudinal clinical-pathologic study of common chronic conditions of old age [20,21]. Study participants are residents of approximately 40 senior housing facilities in the Chicago metropolitan area, including subsidized housing facilities, retirement communities, and retirement homes. Participants in the Rush Memory and Aging Project undergo risk factor assessment and detailed annual clinical evaluations (see below). The study began in 1997 and is ongoing; in 2008, an additional study on neuroeconomics was added. Both parent study and the neuroeconomics studies were approved by the Institutional Review Board of Rush University Medical Center, and informed consent was obtained from each participant following a detailed presentation of the risks and benefits associated with study participation.

At the time of these analyses, 425 participants had completed the neuroeconomics study including measurement of temporal discounting. Of those, 19 met criteria for dementia and were excluded from this study. Further, 18 subjects were excluded because they had missing income. This resulted in a final group of 388 participants (97 males and 291 females), with a mean age of 83.1 years (SD = 6.7; range: 60.0-98.8), a mean education of 15.2 years (SD = 3.1; range: 7–28), and a mean score of 28.1 (SD = 2.0; range: 14–30) on the Mini-Mental State Examination; 94.6% were White and non-Hispanic, 28% reported income lower than $25K, 38% had income between $25K and $50K, and 34% had income over 50K, and the mean number of chronic health conditions reported was 1.8 (SD =1.2; range: 0–5); some of the participants included in these analyses also were included in a prior study of a related but distinct construct of risk aversion [21].

### Assessment of temporal discounting

Temporal discounting was assessed via 7 binary questions, following a standard preference elicitation protocol [22,23]. Participants were asked to choose between an immediate, smaller versus a delayed, larger payment, *e.g.,* “Which do you prefer, that you get $10 in cash right now or $13.50 in a month?” The current payment was fixed at $10 and the delay period was fixed at one month for all questions. Delayed payments ranged from $10.75 to $30, with payment amounts varying across questions ( *i.e.*, they did not escalate in sequence). The Cronbach’s alpha for this measure was 0.86, indicating adequate reliability.

### Clinical and cognitive evaluation

Details of the clinical evaluation have been described previously [20,21]. Briefly, each participant underwent a uniform structured baseline evaluation, including medical history interviews, complete neurological evaluations and neuropsychological examinations. Cognitive function was assessed via a battery of 21 tests [20,21]. Scores on 19 tests were used to create summary indices of global cognitive function and five specific cognitive abilities (i.e., perceptual speed, working memory, episodic memory, semantic memory, and visuospatial function), as previously described [20,21]. To compute the composite measure of global cognitive function, raw scores on each of the individual tests were converted to z-scores using the baseline mean and standard deviation of the entire cohort, and the z-scores of all 19 tests were averaged [20,21]; the same procedure was followed using relevant tests for each of the five cognitive abilities.

Participants with dementia were carefully excluded from these analyses based on annual clinical evaluations that include detailed cognitive testing as well as an in person evaluation by a clinician trained in the assessment of older persons. Clinical diagnoses were performed using a three stage process [20]. First, the neuropsychological tests described above were administered by trained technicians, scored by a computer, and ratings of impairment were assigned based on education-adjusted cut-off scores on 11 cognitive tests commonly used in the assessment of dementia [20]. Second, an experienced neuropsychologist, blinded to subject age, sex, and race, reviewed the results of the cognitive testing including impairment ratings, data on education, sensory and motor deficits, and rendered a clinical judgment regarding the presence of cognitive impairment. Third, diagnostic classification was performed according to standard diagnostic criteria by an experienced clinician blinded to all previously collected data after a review of all available data from that year’s clinical evaluation, including the ratings by the neuropsychologist, MMSE scores and the details of the neurological examination [24,25].

### Assessment of other covariates

Other variables used in the analyses included age, sex, and education (years of schooling completed). In addition, we adjusted for the number of chronic diseases (based on self report of 5 common conditions including hypertension, diabetes, heart disease, cancer, and stroke) and income (based on self-reported data on an evenly-spaced, 10-category ordinal variable) at the study baseline [20]. These covariates were examined because they have been shown to be or likely are related to one’s processing and evaluation of future rewards.

### Data analysis

The discounting factor α was estimated using a well-established hyperbolic function [3,25-28]. However, because there are alternate approaches to estimating discounting, we employed an exponential function that generates k-values for comparison [26,29]. The correlation between the alpha and k-values was 0.99 (p <0.001), suggesting that both approaches would yield similar findings [30]. Thus, we proceeded using the following hyperbolic function

(1) $$V=\frac{A}{1+\alpha D}$$

where *V* represents the discounted value of the future reward *A* at delay *D*_._ The function shows that larger values of α correspond to smaller values of *V*.

Let observed outcome of a trial be denoted by *Y*, the decision to choose a later reward by *Y* = 1 and to choose a current reward by *Y* = 0. We hypothesized that the probability *P*( *Y* = 1) depends on the difference between the discounted future reward *V* and the present reward *C*. The odds of choosing future reward over present reward was formulated as

(2) $$\frac{P\left(Y=1\right)}{P\left(Y=0\right)}=e^{V-C}$$

If V−C=0, this indicates indifference between the current and delayed rewards. If *V - C* is positive, this indicates a preference for the delayed reward with odds greater than 1, and a negative *V - C* suggests a preference for the immediate reward. The discounting rate α could be estimated from Eq (2). To investigate whether α was associated with cognition, we further parameterized the log transformed α as a linear function of variables of interest and applied nonlinear mixed models, adjusted for age, sex, education, income and chronic disease. This approach combines the estimation of α and the hypothesis testing for the association of discounting with cognition in a single model framework. Further, it allows for the estimation of the temporal discounting parameter in 110 participants whose responses were uniform (i.e., choosing either only current or only delayed options across all items). Programming was done in SAS PROC NLMIX [31].

## Results

### Descriptive results

The mean α was 0.018 (SD = 0.022, range: 0.002-0.086, skewness: 2.09, kurtosis: 3.60). For descriptive purposes, Table 1 shows the distribution of participants (overall and separately for those in the top and bottom quartiles of global cognitive function) who chose the delayed payments. Notably, because all delayed payments were larger than the immediate payment of $10, taking the immediate payment (at any point) can be considered an indicator of temporal discounting. When the delayed payment was the smallest ($10.75), only 23% of the overall sample chose the delayed payment. However, this percentage increased as the delayed payment increased, such that by the time the delayed payment reached $30, 92% of the overall sample chose that option. Further, persons with lower cognitive function were less likely to take the larger delayed payments for each question as compared to those with higher cognitive function; for example, among those in the lowest quartile of cognition, 88% took the $30 delayed payment compared to 95% of those in the highest quartile. Finally, it is noteworthy that 344 subjects (89%) answered the questions consistently; that is, once they chose a larger delayed payment, they continued to do so for the rest of the questions with larger delayed payments, even though the questions were not monotonically sequenced.

**Table 1:** Percent of participants who took the delayed, larger payment option for each temporal discounting question

**Delayed payment amount**	**All participants (mean alpha = 0.018, SD = 0.02)**	**High cognition (mean alpha = 0.014, SD = 0.02)**	**Low cognition (mean alpha = 0.022, SD = 0.03)**
10.75	88 (23%)	22(23%)	22(23%)
12.50	183 (47%)	51(53%)	43(45%)
13.50	223 (57%)	67(69%)	42 (43%)
15.00	273 (70%)	73(75%)	59 (61%)
17.50	303 (78%)	80(82%)	68(70%)
20.00	326 (84%)	87(90%)	74 (76%)
30.00	356 (92%)	92(95%)	85 (88%)

*High and low cognition groups represent the top and bottom quartiles on the measure of global cognition, respectively; immediate payment option for all questions = $10.

### Bivariate associations of temporal discounting with demographic and contextual variables and global cognition

Because little is known about the factors associated with temporal discounting in advanced age, we first conducted analyses to examine the bivariate correlations between demographic and contextual factors and global cognition with temporal discounting. In these analyses, global cognitive function were negatively associated with temporal discounting, but age, education, sex, chronic diseases and income were not significantly associated with discounting (Table 2).

**Table 2:** Intercorrelations among cognition and related demographic and contextual variables and discounting

**Variable r**	**Age**	**Sex**	**Education**	**Income**	**Chronic medical conditions**	**Global cognition**	**Temporal discounting (alpha)**
Age		0.064	−0.041	−0.036	0.127*	−0.283**	−0.035
Sex			0.210**	0.221**	−0.097	−0.046	−0.077
Education				0.383**	−0.113*	0.323**	−0.040
Income					−0.096	0.233**	−0.085
Chronic diseases						−0.046	0.043
Global cognition							−0.115*

*indicates p <0.05, ** indicates p <0.001.

### Association of temporal discounting with cognition

First, to directly examine the association between global cognition and discounting, we constructed a mixed effects model adjusted for age, sex, education, income, and chronic diseases. In this fully adjusted core model, global cognitive function was significantly associated with temporal discounting (estimate = −0.45, SE = 0.18, p = 0.015, Table 3), such that a lower level of cognition was associated with greater discounting. Importantly, this association was observed among persons deemed to be free of dementia based on a comprehensive clinical evaluation; however, to ensure that the association of cognition with discounting was not driven by persons at the lower end of cognitive ability, we conducted a sensitivity analysis in which we repeated the core model after excluding persons with a MMSE score of less than 24 (n = 10, 2.6% of the analytic cohort). In this analysis, the association of global cognition with discounting persisted (estimate = −0.54, SE = 0.20, p = 0.007).

**Table 3:** Parameter estimates from the mixed model showing the association of cognition with discounting

**Parameter**	**Estimate**	**SE**	**P-value**
Age	−0.020	0.013	0.137
Sex	−0.318	0.210	0.131
Education	0.012	0.032	0.712
Income	−0.032	0.038	0.400
Chronic medical conditions	0.038	0.075	0.611
Global cognition	−0.446	0.182	0.015

Subsequently, to investigate whether the association of cognition with temporal discounting was of a general nature or reflected domain specific effects, we repeated the model described for global cognition but replaced global cognition with each of five specific cognitive abilities, respectively. As above, all analyses were adjusted for age, sex, education, income, and chronic diseases. Two specific cognitive abilities, perceptual speed (estimate = −0.32, SE = 0.11, p = 0.005) and visuospatial abilities (estimate = −0.33, SE. = 0.13, p = 0.009) were negatively associated with temporal discounting, but the other three domains (*i.e.,* episodic memory, semantic memory and working memory) were not.

## Discussion

In this study, we examined the association of cognitive function, demographic and contextual factors with temporal discounting in a cohort of more than 380 community-based older persons free of dementia. In a mixed effect model adjusted for age, sex, education, income, and chronic diseases, global cognitive function was significantly associated with temporal discounting. Subsequent analyses indicated that this association was driven by perceptual speed and visuospatial abilities. This is the first study that we are aware of to examine whether demographic, contextual and cognitive factors are associated with temporal discounting among community-based older persons. Findings indicate that a lower level of cognitive function is associated with greater temporal discounting among very old community-based persons without dementia.

In recent years, temporal discounting has been the focus of many psychology, economics, behavioral economics, and neuroeconomics studies of younger persons. Findings from these studies have yielded at least two important insights: first, people do not always behave in a dynamically consistent manner (*i.e*., in ways consistent with maximizing utility), as suggested in traditional economic models; and second, temporal discounting is associated with numerous real world economic and health outcomes linked to success and well-being [3,4,32]. Data from highly educated younger persons and adults suggest a negative association between indicators of cognitive ability and temporal discounting, but this association has not been examined in large-scale studies of aging [9-13,33]. This represents a significant gap in knowledge, given that aging is a time when numerous complex and influential life decisions are made just as cognitive function may begin to decline. Consistent with findings reported in younger persons, we found that cognitive ability is negatively related to discounting among older persons. This association was robust to adjustment for other factors such as income and the number of chronic medical conditions.

Some prior studies have investigated whether discounting rates differ in younger versus older persons, and the prevailing suggestion from these studies is that older persons tend to discount less steeply than younger persons; however, the evidence is mixed [15,31,34,35]. We are not aware of studies that have directly examined the association of cognition, demographics and related contextual factors with discounting in community-based persons as old as those studied here. Our findings suggest that the level of cognitive function is the major determinant of an older person’s ability to wait patiently for larger but delayed rewards. Given that temporal discounting has been shown to be associated with many important real world decisions involving the ability to delay gratification (e.g., drug and alcohol use, savings behavior) among younger persons, this finding may have important public health implications for older persons. For example, overwhelming evidence from the real world suggests that older persons are vulnerable to poor and impulsive decision-making. Notably, older persons are selectively vulnerable to fraud and lose more than 2.9 billion dollars annually to financial fraud alone (not counting all other types of fraud). Similarly, older persons frequently make poor financial and health choices with respect to personal matters (e.g., many persons take social security distributions early, often to their own detriment; many also take health supplements not supported by evidence-based medicine). It is conceivable that temporal discounting may be a factor in situations in which older persons make poor decisions and fall prey to scam artists. Future studies are needed to clarify the public health implications of discounting in old age and may suggest policy implications for vulnerable older persons.

To date, most of the existing studies on cognition and discounting have examined indices of general cognitive ability; however, a few focused specifically on working memory because of its relatively strong association with overall intellectual function [12,13]. In one such study, increased working memory load during a discounting task was associated with greater overall discounting [13]. Others have reported similar associations with working memory, and this relation has been interpreted as meaning that active attention to goal specific information and the integration of complex information are important determinants of discounting [12]. In the present study, we found that perceptual speed and visuospatial abilities were related to discounting but working memory was not. The difference between study findings may in part reflect divergent approaches to measuring discounting across studies. Our questions were in some ways simpler than those used previously (*e.g.,* we included fewer and more simply worded questions and held the delay time constant for the larger payments); thus, the tasks used in earlier studies were more difficult and may have demanded more working memory than that employed in this study. Further, perceptual speed involves the ability to efficiently process information and make mental comparisons, and it is intuitive that these abilities are associated with the ability to advantageously decide between an immediate, smaller payment and a delayed, larger one. Moreover, perceptual speed, like working memory, is considered a component of the broader domain of executive function, which includes higher-level cognitive processing and abstract reasoning abilities that support the ability to wait for a delayed reward.

The finding that visuospatial abilities were associated with temporal discounting was unexpected; however, some data suggest that visuospatial deficits may precede the onset of dementia in old age and are associated with a deterioration in independent activities such as driving) [36]. Somewhat akin to driving (albeit less complex), temporal discounting tasks require processing relational associations and estimating reward size and magnitude, cost benefit ratios, and timing. Thus, it is possible that persons with poorer visuospatial ability were less able to efficiently identify relevant patterns (*i.e*., delayed payments are larger) and integrate multiple sources of information. However, additional research is needed to clarify the potential association of visuospatial abilities with discounting.

Strengths of this study include the comprehensive evaluation of cognition and temporal discounting in a fairly large cohort of community-based older persons. This enabled us to examine the association of global cognition and five specific cognitive abilities with temporal discounting among persons carefully evaluated and determined to be free of dementia. Limitations included the volunteer cohort, which may limit the generalizability of findings, and the single assessment of discounting using a series of hypothetical questions. Although a task involving actual monetary payment may be preferable, prior studies using hypothetical tasks like that used here have shown that the tendency to discount future rewards (or the inability to delay gratification) is associated with numerous psychological problems including gambling, substance abuse, depression and anxiety, as well as poorer academic, occupational and financial outcomes [3,4,6,8,10,22,27,37]. These data provide strong support for the ecological validity of measures such as that used here. Longitudinal study would enable us to examine the dynamic changes that may occur in both cognition and temporal discounting in aging. Future studies are needed to investigate whether and how the associations of cognition and age with temporal discounting change over time with advancing age and to clarify the association of discounting with decision-making and health outcomes in old age.

## Conclusion

Temporal discounting is an important determinant of many important real world behaviors including financial, occupational and health outcomes, but there are little data on temporal discounting among older persons. The present findings indicate that a lower level of cognitive function is associated with greater temporal discounting among community-based older persons without dementia. These findings may have implications for efforts to improve decision making in advanced age.

## Competing interests

The authors have no disclosures to report.

## Author contributions

PAB, DAB, and DIL were involved in the study concept and design, acquisition of subjects and/or data, analysis and interpretation of data, and preparation of manuscript. LY, ES, RSW and ASB were involved in the analysis and interpretation and preparation of the manuscript. All authors read and approved the final manuscript.

## Sponsor’s role

The sponsor (NIA) provided funding but had no role in the design, methods, subject recruitment, data collections, analysis or preparation of the paper.

## Pre-publication history

The pre-publication history for this paper can be accessed here:

http://www.biomedcentral.com/1471-2318/12/48/prepub
